# Trauma Disrupts Reinforcement Learning in Rats—A Novel Animal Model of Chronic Stress Exposure

**DOI:** 10.3389/fnbeh.2022.903100

**Published:** 2022-05-17

**Authors:** Tomasz Bielawski, Jarosław Drapała, Paweł Krowicki, Bartłomiej Stańczykiewicz, Dorota Frydecka

**Affiliations:** ^1^Department of Psychiatry, Wrocław Medical University, Wrocław, Poland; ^2^Department of Computer Science and Systems Engineering, Faculty of Information and Communication Technology, Wrocław University of Science and Technology, Wrocław, Poland; ^3^Department of Laser Technologies, Automation and Production Management, Faculty of Mechanical Engineering, Wrocław University of Science and Technology, Wrocław, Poland

**Keywords:** reinforcement learning, trauma, PTSD, predator odor, chronic stress

## Abstract

Trauma, as well as chronic stress that characterizes a modern fast-paced lifestyle, contributes to numerous psychopathologies and psychological problems. Psychiatric patients with traumas, as well as healthy individuals who experienced traumas in the past, are often characterized by diminished cognitive abilities. In our protocol, we used an animal model to explore the influence of chronic trauma on cognitive abilities and behavior in the group of 20 rats (Rattus norvegicus). The experimental group was introduced to chronic (12 consecutive days) exposure to predator odor (bobcat urine). We measured the reinforcement learning of each individual before and after the exposition *via* the Probabilistic Selection Task (PST) and we used Social Interaction Test (SIT) to assess the behavioral changes of each individual before and after the trauma. In the experimental group, there was a significant decrease in reinforcement learning after exposure to a single trauma (Wilcoxon Test, *p* = 0.034) as well as after 11 days of chronic trauma (Wilcoxon-test, *p* = 0.01) in comparison to pre-trauma performance. The control group, which was not exposed to predator odor but underwent the same testing protocol, did not present significant deterioration in reinforcement learning. In cross-group comparisons, there was no difference between the experimental and control group in PST before odor protocol (U Mann-Whitney two-sided, *p* = 0.909). After exposure to chronic trauma, the experimental group deteriorated in PST performance compared to control (U Mann-Whitney Two-sided, *p* = 0.0005). In SIT, the experimental group spent less time in an Interaction Zone with an unfamiliar rat after trauma protocol (Wilcoxon two-sided test, *p* = 0.019). Major strengths of our models are: (1) protocol allows investigating reinforcement learning before and after exposition to chronic trauma, with the same group of rats, (2) translational scope, as the PST is displayed on touchscreen, similarly to human studies, (3) protocol delivers chronic trauma that impairs reward learning, but behaviorally does not induce full-blown anhedonia, thus rats performed voluntarily throughout all the procedures.

## Introduction

Throughout life, the environment puts numerous stressors on every living organism. In humans, extreme stress (trauma) captures a range of severe adverse experiences, such as physical, sexual, or emotional abuse, neglect, parental death, bullying, or omission by caregiver during childhood. Trauma contributes to the development of numerous mental disorders such as posttraumatic stress disorder (PTSD), anxiety disorders, schizophrenia, personality disorders, mood disorders (Jansen et al., 2016; Misiak et al., 2017). It is estimated that prevalence of PTSD reaches 7% in general population (McLaughlin et al., 2015), while in subgroups exposed to severe psychological trauma numbers are even more prominent, for example, 10% of US veterans meet criteria of PTSD (Mota et al., 2016) as well as 60% minor refugees in Germany that sought general medical treatment (Veeser et al., 2021). In the general population, only a small proportion of individuals with a positive history of traumatic events develop full-blown PTSD (Breslau, 2009). Trauma affects cognitive abilities (Petkus et al., 2018; Aas et al., 2019), disrupts the immune system (Mehta et al., 2020), causes structural changes in the brain (Assogna et al., 2020), affects the severity of symptoms among those with mental disorders (Duhig et al., 2015; Ay and Erbay, 2018; Bailey et al., 2018). Chronic stress, defined as an exposition to a series of stressful or potentially traumatic events, characterizes a modern, fast-paced western lifestyle (Matosin et al., 2017). Chronic stress turns out to be closely related to numerous health issues: obesity, diabetes, mental disorders, psychological deficits, substance dependence (Sinha, 2008; Farag and Gaballa, 2011; Misiak et al., 2017; Bielawski et al., 2019). All are major epidemiological health concerns that generate enormous public cost (Simon et al., 2006; Farag and Gaballa, 2011; Laramée et al., 2013; Masodkar et al., 2016). The purpose of this study is to present a novel protocol to examine cognitive impairment in reinforcement learning as chronic trauma progresses. We use a simplified Probabilistic Selection Task (PST) to approximate our model to human studies. In humans, experimental studies of PTSD, chronic stress, and trauma are limited. Therefore, our research is to explore the translational scope of PTSD studies in rodents. We want to test whether rats will perform voluntarily while exposed to chronic trauma. If so, our aim is to study rats’ ability to learn the PST protocol, as well as their ability to adapt to a system, where interaction with a touchscreen is related to reward collection. Our procedure examines reward learning before and after exposure to chronic trauma, with the same group of rats. This approach allows us to measure cognitive disruptions as the trauma progresses. We hypothesize that rats exposed to trauma will perform poorer in PST, in comparison to their performance before exposure to chronic trauma. Moreover, we want to explore whether a single exposure to trauma will affect cognitive functioning. Furthermore, we hypothesize that traumatized individuals will be less socially oriented during Social Interaction Test (SIT), compared to the control.

## Materials and Methods

### Theoretical Background

#### Trauma, Cognition, and Chronic Stress Rationale

Medically oriented understanding of psychological trauma is strictly related with PTSD diagnosis (Yehuda, 1998), while in psychoanalytic approach trauma is a powerful stimulus, that breaches one’s psychological defense mechanisms, and induces experience of helplessness (Rothgeb, 1971). In both definitions trauma is an extreme stress, that is beyond one’s ability to cope with. An abundant literature presents negative impact of trauma on cognitive functions in patients with psychosis (Lysaker et al., 2001; Schenkel et al., 2005; Shannon et al., 2011) and among healthy individuals who experienced trauma in the past (Majer et al., 2010; Vasilevski and Tucker, 2016; Petkus et al., 2018). Trauma and prolonged (chronic) stress activate the hypothalamic-pituitary-adrenal (HPA) axis *via* the rise of corticosteroids, activate the endocannabinoid system, and indirectly affect dopamine bursts in the striatum and medial prefrontal cortex (Joëls et al., 2012; Bielawski et al., 2019). Different regions of the brain (for example hippocampus, amygdala, medial prefrontal cortex, hypothalamus) involved in stimulus recognition, memory, and learning are affected by increased detrimental corticosteroids rise during chronic or acute stress (Pruessner et al., 2017; Bielawski et al., 2019). The neurobiology of trauma and its impact on cognitive abilities is complex, and studies in human subjects have certain limitations. Thus, several animal models have been developed to assess symptoms associated with exposure to trauma and the development of PTSD (Whitaker et al., 2014; Harro, 2018; Planchez et al., 2019). The Diagnostic and Statistical Manual of Mental Disorders version 5 (DSM-V) delivered by the American Psychiatric Association (APA) presents four clusters of symptoms of PTSD: intrusive recollection of the original traumatic event, avoidance of trauma-related reminders, negative changes in cognition and mood, and alterations in arousal or reactivity, each of which must start or be significantly exacerbated after exposure to the traumatic event (Roehr, 2013). The variety of animal models put its focus on different aspects of PTSD symptomatology, such as contextual avoidance (Albrechet-Souza et al., 2020), changes in arousal and reactivity (Knox et al., 2012), and behavior alterations (Krishnan et al., 2007). These models measure different parameters after the exposition to stress. Our approach is to measure cognitive and behavioral parameters as chronic trauma progresses. That way, an animal model gives us an opportunity to expose rats to chronic stress, as we measure their cognitive functions simultaneously. Chronic stress lacks a clear definition, but most authors agree that it is an exposition to a series of intense, potentially traumatic experiences or involvement in prolonged stress situations that leads to psychopathologies and/or adverse medical conditions (Matosin et al., 2017). Chronic stress is widely used in animal models of anxiety disorders, depression, and PTSD (Saavedra-Rodríguez and Feig, 2013; Reber et al., 2016; Wang et al., 2021). In humans, prolonged stress is an important factor in etiopathology of different mental disorders (Matosin et al., 2017; McEwen, 2017; Ross et al., 2017), for example chronic stress can induce mild PTSD symptoms in humans (Davidson and Baum, 1986). Stress influences the ability to learn from rewards among those with a familial predisposition to psychosis and individuals with major depressive disorder (Reinen et al., 2021). Furthermore, chronic stress induces hyper inflammation, thus being discussed to enhance susceptibility to infectious diseases such as COVID-19 (Lamontagne et al., 2021), or mental diseases linked to immune system dysregulations (Dennison et al., 2012). Chronic exposure to trauma is particularly harmful; many individuals repeatedly exposed to traumatic events carry a heavy burden of psychopathologies (Sharhabani-Arzy et al., 2003; Éthier et al., 2004; Salcioglu et al., 2017). In our experiment, we expose male Wistar Rats to chronic trauma for 12 consecutive days. In the literature, there are animal models of PTSD that reveal alteration in cognitive performance, although they often apply a single prolonged stress procedure (George et al., 2015). Indeed, single exposure to predator odor is sufficient to induce trauma (Albrechet-Souza and Gilpin, 2019), but our goal is to mimic chronic stress, thus our protocol’s prolonged exposure to stressful stimulus with parallel cognitive examination.

#### Probabilistic Selection Task and Social Interaction Test

In humans, the Probabilistic Selection Task (PST) was shown to be associated with dopaminergic effects on learning (Frank et al., 2007). Positron emission tomography and functional magnetic resonance imaging studies showed that reinforcement-based decisions are associated with signaling in the striatum and prefrontal cortex (Jocham et al., 2011; Kasanova et al., 2018). Furthermore, PST was used to assess learning deficits among those with PTSD (Myers et al., 2013). During PST, participants are presented stimulus pairs and learn to choose one of them. After each choice, probabilistic feedback follows the choice to indicate whether it was correct or incorrect. PST (and its different variants) are widely used in animal studies—in rodents stimulus selection is most often recorded *via* nose poke in aperture (Amitai et al., 2014) or by pressing the lever (George et al., 2015; Seib et al., 2020), while in humans selection is usually done *via* tap on a touchscreen or pressing a button on a keyboard (Frank et al., 2004).

The Social Interaction Test (SIT) is a popular method to assess levels of anxiety, social interaction, locomotor activity, and arousal in rodents (File and Seth, 2003). In our experiment, an examined rat is introduced into the test box with a tunnel, open field arena, and Interaction Zone with unfamiliar rat. Examined rat behavior is monitored; time spent in different parts of the test arena, number of droppings, or freezing behavior. Our model explores cognitive changes among Wistar Rats through the PST, as well as anxiety level and social interaction through the Social Interaction Test. We used SIT procedure similar to the one in social defeat experiments (Golden et al., 2011; Toyoda, 2017).

#### Predator Odor as Traumatizing Factor

In our study, we use an animal model with predator odor exposure that produces behavioral, physiological, and molecular alterations that recapitulate many of the same alterations observed in PTSD patients (Cohen et al., 2012). We use bobcat urine as a stressor, it is a well-established model used in a series of studies done by Gilpin and colleagues (Albrechet-Souza and Gilpin, 2019). Bobcat urine contains the biogenic amine 2-phenylethylamine, which activates specific receptors within the rodent olfactory cortex, the trace amine-associated receptor 4 (TAART4), and can induce avoidance behavior in rats and mice (Ferrero et al., 2011). Furthermore, bobcat urine activates the amygdala-piriform transition area, which is responsible for increases in circulating stress hormones (Kondoh et al., 2016). In 1993, Yehuda and Antelman developed 5 criteria that animal models must meet, to parallel PTSD-related phenotypes: (1) Even a brief stressor should be able to induce biological and behavioral sequelae of PTSD, (2) The stressor should be able to produce PTSD-like sequelae in a dose-dependent manner, (3) Stressors should produce biological alterations that persist over time or become more pronounced with passage of time, (4) The stressor should induce biobehavioral alterations that have the potential for bidirectional expression, (5) Interindividual variability in response to a stressor should be present either as a function of experience, genetics, or an interaction of the two (Yehuda and Antelman, 1993). Studies done with bobcat urine meet most of those criteria (Albrechet-Souza and Gilpin, 2019), and are well discussed in the context of animal PTSD model (Albrechet-Souza et al., 2020, 2021). Taking the literature mentioned above, we feel confident using this type of traumatizing stimulus in our protocol.

### Subjects

In our procedure, we used male Wistar Rats (Animal Research Center, Wrocław Medical University, PL) in a total number of 26 individuals (*n* = 26), although 20 individuals were included in our experiment (*n* = 20). Rats arrived at the age of 39–42 days, weighing 210–245 g at the day of arrival, were submitted to a handling period (7 days), and then entered P0. Six individuals did not meet the criteria to enter the P1, and were excluded during P0. Excluded animals either: (1) did not learn the tapping procedure throughout phase 0 or (2) presented freezing behavior during 3 consecutive days. Due to housing conditions and experimental procedure, the exclusion of a rat resulted in the exclusion of its cotenant. Therefore, even though *n* = 3 rats met the exclusion criteria, the total sum of *n* = 6 individuals was excluded.

A random group of rats (*n* = 10) participated as a control group, the second group (*n* = 10) participated as an experimental group (*n* = 10). Rats were pair housed on a non-reversed 12 h/12 h light/dark cycle (lights off at 7 p.m.). All behavioral tests were constructed during the light period. Rats had *ad libitum* access to food (dry pellets) and water.

The experiment was conducted in accordance with the NIH Guide for the Care and Use of Laboratory Animals. All procedures were approved by the Local Ethics Committee for Animal Experiments, Hirszfeld Institute of Immunology and Experimental Therapy, Polish Academy of Sciences, Wrocław, Poland.

### Testing Chambers

**FIGURE 1:**
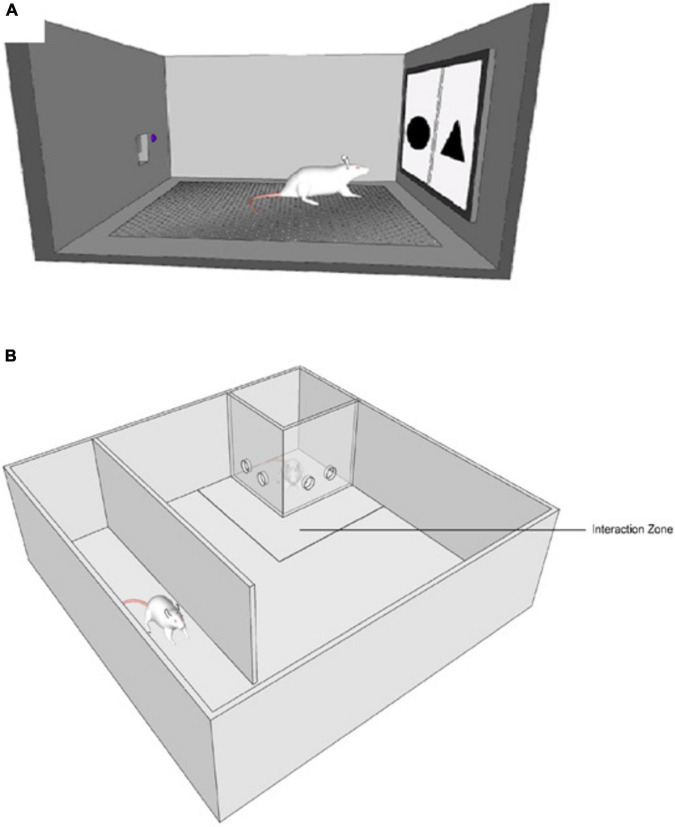
**(A)** Probabilistic Selection Task testing chamber. **(B)** Social Interaction Test testing chamber.

The PST chamber was part of the device built by our team to measure PST in rats. It had a perforated metal floor that allowed animals to move freely and comfortably. Under the perforated floor there was a compartment where a sponge with odor could have been placed. The walls and floor of the chamber were easy to sanitize and safe for the animals to explore. The front wall had a hole, where a touchscreen apparatus displayed stimuli. Opposite the front wall, there was a feeder and a diode. Feeder was the place where rewards was delivered, a diode signaled when reward was about to be delivered (see Figure 1A).

SIT chamber was constructed from polyvinyl chloride (PCV) and Plexiglas. The main structure was a square 90 × 90 × 40 cm (length × width × height). Inside, there was a PCV wall 70 × 30 cm (length × height) that formed a tunnel. Furthermore, two additional transparent Plexiglas walls (20 × 30 cm) formed a closed space in one of the corners, where a new and unfamiliar rat was trapped (see Figure 1B). The 25 cm from plexiglas walls was marked as an “interaction Zone.”

### Procedure

**FIGURE 2:**
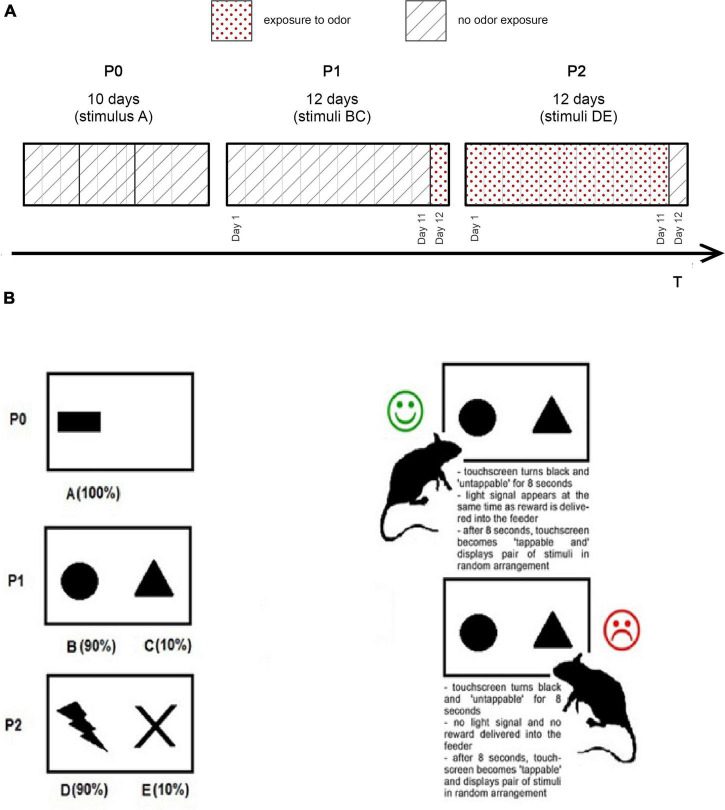
**(A)** Experiment time schedule. **(B)** Probabilistic Selection Task procedure. P0, Phase 0; P1, Phase 1; P2, Phase 2.

PST- one pair of stimuli is presented in random order arrangement (left of right side of the screen) (see Figure 2B). Rats learned to choose one pair. Feedback was probabilistic; it means that in BC trials, a choice of stimulus B results in 90% positive feedback (10% negative feedback), while choice of stimulus C results in 90% negative feedback (10% positive feedback). Feedback follows the choice to indicate if it was correct (reward) or incorrect (punishment). The correct choice resulted in reward—a drop of sweet protein shake (Strawberry Nutridrink Protein, NUTRICIA, Poland). Incorrect choice resulted in punishment—lack of reward. The touchscreen was 26.5 cm width × 17 cm height and “tappable”—nose poke, strike, or touch with paw resulted with stimulus selection. When the stimulus was selected, the touchscreen went black for 8 s and a reward was delivered to the feeder, simultaneously with a light signal. After 8 s, the touchscreen displayed a randomly arranged pair of stimuli again.

SIT was performed twice throughout the experiment, P1 Day 6 and P2 Day 6. Examined individual was placed at the beginning of the tunnel. The session lasted 10 min and was videotaped.

### Experimental Design

The rats were subjected to 1 week of handling before the phase 0. During handling sessions, rats were exposed to a sweet liquid, to adapt with sweet reward and feeder mouthpiece. An experiment consisted of 3 phases: phase 0 (P0), phase 1 (P1), and phase 2 (P2) (see Figure 2A). Each rat was examined *via* PST in the testing chamber once every day. Our protocol is a variation of the autoshaping task described by Horner et al. (2013).

#### Phase 0

P0 lasted 10 days and was designed to teach each animal the experimental procedure. During Day 1–3, the paired rats (according to the pair housing) were placed in the testing chamber to accommodate. The rats were able to explore the chamber for 20 min and collect rewards. During the first 6 days, the touchscreen displayed one visual stimulus on the left or right side (see Figure 2B). During the first 6 days, the rest of the touchscreen was “untappable”—there was no selection when tap occurred outside the stimulus sector. From 4 to Day 10, the rats were placed in the testing chamber separately, 10 min each.

Throughout Day 7–10 the stimulus was randomly displayed on the left or right side of the touchscreen, although the whole surface of the touchscreen was tappable. Tap delivered within the sector outside of the stimulus resulted in punishment—touchscreen went black for 8 s, no reward was delivered into the feeder. After 8 s, the touchscreen displayed the stimulus again randomly (left or right).

#### Phase 1

P1 lasted 12 days. Throughout P1, a pair of stimuli (B and C) was used in PST (see Figure 2B). Each animal was placed in the testing chamber for 20 min or until the session was completed. After each session, the testing chamber was thoroughly cleaned with disinfectant. The last day (P1 day 12) animals were exposed to predator odor, a sponge soaked with 3 ml of bobcat urine (Lynx rufus; Maine Outdoor Solutions, Hermon, ME, United States) was placed on the testing chamber floor. In the control group, sponges were not soaked with bobcat urine.

#### Phase 2

P2 lasted 12 days. Throughout P2, a pair of stimuli (D and E) was used in PST (see Figure 2B). Each animal was placed in the testing chamber for 20 min or until the session was completed. After each session, the testing chamber was thoroughly cleaned with disinfectant. Throughout P2, a sponge soaked with bobcat urine (Lynx rufus; Maine Outdoor Solutions, Hermon, ME, United States) was placed under the testing chamber floor. The Last day (P2 Day 12) the animals were not exposed to predator odor. Control rats are treated identically to rats exposed to odors, but the sponges were not soaked with bobcat urine.

### Data Collection

During the experiment, the rats performed PST once a day. Each session had 20 trials, the sessions ended when the last trial was completed or when 20 min passed. P1 and P2 lasted 12 days; we measured performance of each rat during 1, 11, and Day 12 (see Figure 2A). During those days, we recorded the number of wins (rewards delivered) and loses (punishment received).

In the experimental group, Day 1 was the day when a novel pair of stimuli was presented for the first time. Day 12 was the last day with a pair of known stimuli, but with changed environmental factors (odor or no odor exposure). Thus, P1 Day 1 was the first day when stimuli BC were displayed during PST, without exposure to odor. Day 11 of P1 was the day when stimuli BC were displayed without odor for the last time. P1 Day 12 was the day when BC stimuli were displayed for the last time, but this time with odor exposure. Accordingly, Day 1 ofP2 was the first day when stimuli DE were displayed during PST sessions, with odor exposure. P2 Day 11 was the day when DE stimuli were displayed with odor for the last time. P2 Day 12 was the day when stimuli DE were displayed for the last time, but this time without exposure to odor (see Figure 2A).

### Behavioral Analysis

Video records were scored by the independent observer, who used stopwatch to measure the time spent in the Interaction Zone of each rat. Interaction Zone was outlined on the SIT floor. Crossing the line with hind limbs was considered as entry into the Interaction Zone.

### Statistical Analysis

Analysis and interpretation of behavioral data acquired *via* PST is commonly aided by different variants of theoretical Q learning models (Frank et al., 2004; Frank, 2006; Frank and Claus, 2006; Brown et al., 2018; Kane et al., 2019; Metha et al., 2020). In this way, the research hypothesis is expressed as a set of mathematical equations that govern the analysis of the data. However, the theoretical model introduces its own assumptions and requires advanced routines to adjust the model to the dataset, which may bias the results in an unpredictable manner. Since our study involves a small amount of data, we decided to rely only on directly measurable variables, making the analysis model independent; thus, we present our data without a computational framework.

The test score of each individual was calculated during Days 1, 11, and 12—ratio of the gained rewards to all trials taken that day

$$T\,s\,c\,o\,r\,e_{D\,x}=\frac{N\,r\,e\,w\,a\,r\,d\,s_{D\,x}}{N\,r\,e\,w\,a\,r\,d\,s_{D\,x}+N\,l\,o\,s\,s\,e\,s_{D\,x}},$$

where *Nrewards*_*Dx*_ is the total number of rewards received during day X (Dx) and *N losses*_*Dx*_ is the total number of punishment received during day X (Dx).

Then, we calculated the WinRatio of each individual for P1 and P2. WinRatio was a difference between Test score Day 11 and Test Score Day 1:

$$W\,i\,n\,R\,a\,t\,i\,o=\;\,\left[\frac{N\,r\,e\,w\,a\,r\,d\,s_{D\,11}}{N\,r\,e\,w\,a\,r\,d\,s_{D\,11}+N\,l\,o\,s\,s\,e\,s_{D\,11}}\right]-\;\,\left[\frac{N\,r\,e\,w\,a\,r\,d\,s_{D\,1}}{N\,r\,e\,w\,a\,r\,d\,s_{D\,1}+N\,l\,o\,s\,s\,e\,s_{D\,1}}\right]$$

**FIGURE 3:**
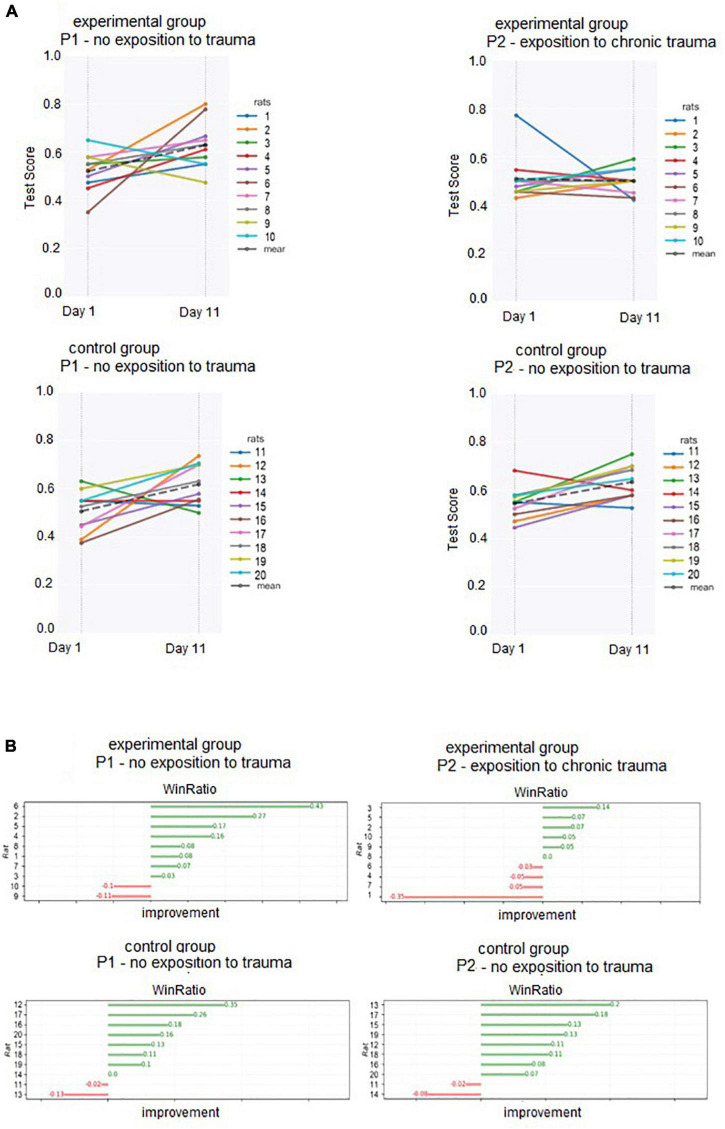
**(A)** Test scores of each individual (obtained in Days 1 and 11) used to calculate P1 and P2 WinRatios. **(B)** Numerical representation of the overall WinRatio of each individual in P1 and P2.

**FIGURE 4:**
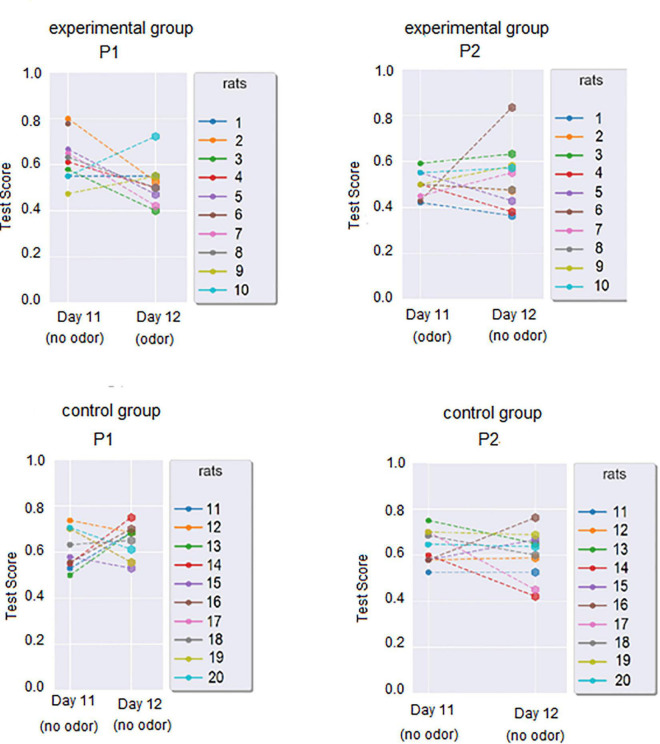
Test scores gained during PST in the last 2 days of each phase. For the experimental group, P1 Day 11 was the day with known stimuli in PST and no odor, but the P1 Day 12 was the first exposure to odor, with stimuli known from previous days. Inversely, P2 Day 11 was the day with known stimuli in PST with odor, while P2 Day 12 was the day with known stimuli in PST, but without odor exposure.

Day 1 and Day 11 test scores (used to calculate individual WinRatios) are presented in Figure 3A. Each rat’s P1 WinRatio and P2 WinRatio is presented numerically in Figure 3B. Days 11 and 12 test scores are presented in Figure 4.

Due to a low number of rats and possibly non-normal distribution of variables, we used non-parametric statistical tests. To compare the performance of PST during P1 and P2 of the same rat, we used the Wilcoxon two-sided test. In cross-group comparisons, the U-Mann-Whitney two-sided test was used. Behavioral results were analyzed using the Wilcoxon two-sided test to compare times each rat spent in an Interaction Zone before and after the trauma, U-Mann-Whitney two-sided test was applied for cross-group comparisons. The statistical significance level was established at *p* < 0.05.

Statistical analysis was performed using the scipy.stats library belonging to the Python programming language ecosystem.^1^

## Results

**FIGURE 5:**
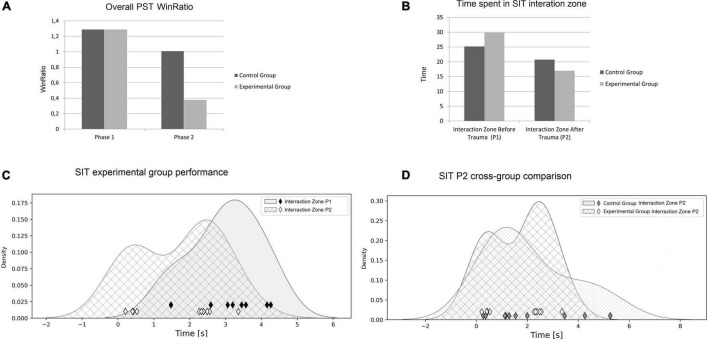
**(A)** Rewards collected in PST throughout the entire experiment. **(B)** Time spent in an Interaction Zone in SIT, comparison between two groups of rats. **(C)** Time spent in an Interaction Zone during SIT (experimental group). Raw measurements data are drawn as diamonds. For easy visual groups comparison we provided kernel density estimates of Probability Density Functions (Silverman, 2018). The experimental group spent significantly more time in an Interaction Zone before trauma exposure (P1). Furthermore, exposure to trauma (P2) induced a bimodal data distribution that has not occurred in P1. **(D)** The experimental group P2 times compared to the control group P2 times. There is no significant differences in group comparisons (*p* = 0.91), but this may be due to bimodality that characterizes post stress-performance of the experimental group.

With each individual’s WinRatio for P1 (no odor) and P2 (with odor), we compared reinforcement learning before (P1) and after (P2) exposure to trauma in the experimental group, as well as reinforcement learning in the control group (see Figure 3B). In the experimental group, WinRatio during P1 was significantly greater than during P2 (Wilcoxon test, *p* = 0.01). In the control group, there was no significant difference in WinRatio between P1 and P2 (Wilcoxon Two-Sided Test, *p* = 0.73). In cross-group comparisons, the control group had a higher P2 WinRatio than experimental group P2 WinRatio (U Mann-Whitney Two-sided, *p* = 0.0005). There was no significant difference between the experimental P1 WinRatio and the control P1 WinRatio (two-sided Mann-Whitney U, *p* = 0.909). In general, both groups WinRatios are presented in Figure 5A.

The test score was calculated for Day 12 in P1 and P2 (see Figure 4). In the experimental group, the P1 Day 12 Test score was significantly worse than the P1 Day 11 test score (Wilcoxon Test, *p* = 0.034). In the control group, the P1 Day 12 Test score was similar to the P1 Day 11 test score (Wilcoxon two-sided, *p* = 0.0557). In the experimental group, day 12 P1 and day 12 P2 Day 12 did not differ (Wilcoxon, two sides, *p* = 1.0). In the control group, Day 12 P1 and Day 12 P2 Day 12 did not differ significantly (Wilcoxon two-sided, *p* = 0.314). In cross-group comparisons, the experimental group P1 Day 12 test score was significantly lower than in the control group (U Mann-Whitney, *p* = 0.003).

Figure 5B present differences in the time spent in an Interaction Zone of SIT in P1 and P2. The experimental group spent significantly more time in the Interaction Zone before trauma (P1) compared to time spent in Interaction Zone after predator odor (P2) (Wilcoxon two-sided test, *p* = 0.019). In the control group, there were no significant differences in the time spent in an Interaction Zone during P1 and P2 (Wilcoxon two-sided test, *p* = 0.43). During P1, the experimental group spent similar time in an Interaction Zone to the control group (Wilcoxon two-sided test, *p* = 0.038). Similarly, cross-group comparisons did not reveal differences between both groups in time spent in an Interaction Zone during P2 (Wilcoxon two-sided test, *p* = 0.91) (see Figure 5B).

## Discussion

In our study, we examined reinforcement learning (through PST) before and after trauma and compared obtained results with the untraumatized control group. In the experimental group, exposure to chronic trauma (which occurred every day for 12 consecutive days) significantly reduced the ability to perform on PST. The decline in cognitive ability was significant immediately after the first exposure to trauma, although this result is not surprising. Previous findings indicate that single exposure to predator odor is sufficient to induce a behavioral and physiological response such as avoidance (Albrechet-Souza and Gilpin, 2019) or an increase in alcohol intake (Edwards et al., 2013). To our knowledge, we are the first to report a decline in reinforcement learning immediately after exposure to predator odor. We did not find a significant improvement in PST performance 1 day after the odor removal. This result stays in line with studies reporting that the consequences of odor exposure persist weeks after initial exposure (Albrechet-Souza and Gilpin, 2019; Schreiber et al., 2019). To the best of our knowledge, our study is the first to examine rodent cognitive abilities *via* PST before and after exposure to predator odor. Moreover, our study confirmed bobcat urine utility as a traumatizing factor, as it significantly affected cognitive abilities, and influenced social behavior among rats exposed to odor.

Overall, the control group performed significantly better in P2 of the experiment. During that period of time, the experimental group was chronically exposed to predator odor. This enforced vigilance and anxiety among rats, which resulted in significant deterioration in PST performance, even though neither punishment nor the physical threat was ever delivered. There are numerous animal models with severe physical punishments, for example foot shock, underwater trauma, restrained stress (Whitaker et al., 2014). Our model is not one of them; the punishment was the lack of the reward. In humans, there are protocols that expose subjects to the possibility of punishment that is never delivered. These studies confirm that anticipation stress reduces reward sensitivity, reward responsiveness (Bogdan and Pizzagalli, 2006; Berghorst et al., 2013) and generally impairs reinforcement learning (Cavanagh et al., 2011). Interestingly, it is hypothesized that stress-susceptible individuals may be more vulnerable to punishment than reward collection (Berghorst et al., 2013). In that case, our protocol (which did not present tangible punishment) may have been less perceptive to those subjects. On the other hand, literature implies that individuals who are less stress-susceptible may be more vulnerable to reward collection than to punishment deliverance (Cavanagh et al., 2011), an observation that validates our approach. This distinction in susceptibility is discussed to be related to striatal dopamine levels, which are known to guide decision making in relation to learning from positive and negative stimuli. Patients with pharmacologically elevated dopamine levels learn better from rewards in PST, compared to those with reduced dopamine levels, who learn better to avoid punishment in PST (Frank et al., 2004). Thus, we hypothesize that the experimental group performed in PST poorer in P2, due to disrupted dopamine levels in the striatum. This implies decline in PST was related to the disruption in reward learning circuits. In humans, exposure to chronic stressors results in blunted ventral striatal (VS) neural activity during reward processing in healthy individuals (Nikolova et al., 2012), as well as in those with PTSD (Mehta et al., 2020). The prominent function of dopaminergic VS neurotransmission in reinforcement learning was confirmed in human positron emission tomography studies that mark right caudate and VS as motivational centers of engagement in activity that brings profit (Kasanova et al., 2017). Stress-related blunted dopaminergic neurotransmission results in overall worse performance in PST, a phenomenon that was observed among individuals with a familial risk of psychosis. Thus, disruption in VS is often symptomatically related to anhedonia, depression, and motivation deficits in both humans and animals (Malone et al., 2009; Roesch et al., 2009; Corral-Frías et al., 2015). We hypothesize that chronic trauma, induced in the experimental group, reduced dopamine level in VS that decreased the performance of experimental rats in PST P2. Our protocol delivered chronic trauma that compromised reward learning, but behaviorally did not induce full-blown anhedonia. We believe that is an important advantage of our model—rats perform voluntarily, which facilitates measurement of cognitive and behavioral deficits in rodents.

Our results are in agreement with studies that indicate deterioration in cognitive abilities among those exposed to trauma. Schizophrenia patients with a history of trauma exhibit poorer cognitive functioning in terms of memory, executive functions, attention, concentration, and mental speed (Misiak et al., 2017). Computational studies present altered reinforcement learning in veterans with diagnosed PTSD, indicating alteration in reward and punishment perception and valuation (Myers et al., 2013; Brown et al., 2018). Moreover, individuals with PTSD have increased sensitivity to an unexpected outcome during PST (Brown et al., 2018). To our knowledge, this phenomenon has not been validated in animal models, although we believe our protocol may be in use in further research of this topic. If this mechanism of overreaction to an unexpected outcome occurs in the rodent model of trauma, it could have explained the deterioration in learning during P2. We hypothesize that our traumatized subjects were more susceptible to unexpected punishment in P2—as feedback was probabilistic, rewarding stimulus rarely delivered punishment. To test this hypothesis in the future, our protocol needs to be recreated using a computational model.

In SIT, the experimental group proved to be less socially oriented in P2, in comparison to P1—after trauma, rats spent less time in an Interaction Zone with an unfamiliar rat. In humans, chronic trauma influences social interactions, especially in children. Youngsters exposed to chronic traumatic stress present substantial difficulties in constructing relationships. They have troubles in interactions with other children as they often display avoidant symptoms, present inadequately sensitive flight/fight responses, respond to minor stressors by freezing (Streeck-Fischer and van der Kolk, 2000). In another study, adults with PTSD after 2-years of military deployments presented avoidance behavior, social withdrawal, had less positive engagement in relation with their families during post-deployment reengagement (Brockman et al., 2016). In rodent, chronic social defeat model reveals significant decreases in interpersonal interactions after exposure to trauma (Venzala et al., 2012). We believe results obtained during our experiment stays in line with these reports. We hypothesize that it may be related to dopamine disruption, since social behavior in rodents has been shown to be strongly dependent on neural activity in the ventral tegmental area (VTA) of the brain (Chaudhury et al., 2013). Dopamine neurons in VTA project signals to different structures in the striatum (for example, nucleus accumbens) as the well as amygdala or medial prefrontal cortex. Manipulation in neural projection dynamics of VTA influences social interactions in rodents (Gunaydin et al., 2014); therefore, we hypothesize that our trauma protocol disrupts dopamine levels in the midbrain, which results in reduced social behavior after exposition. In the control group, there was no significant difference in SIT performance in P1 and P2, as rats were not exposed to trauma. Similarly, there was no significant difference in the performance of the experimental group P1 and the control group P1 in SIT, as none of the subjects was exposed to predator odor. Although the experimental group spent significantly more time in an Interaction Zone during P1 in comparison with P2, statistical analysis does not reveal differences in time spent in an Interaction Zone between experimental group and the control during P2. This result is inconclusive—two factors have to be taken into consideration. First, a performance difference was observed (see Figure 5B), but we cannot support this with statistical verification, probably due to the small number of rats tested. Second, the distribution of the time spent in an Interaction Zone among rats exposed to trauma was bimodal (see Figure 5C). This makes the verification of this particular result ambiguous, as a control group did not present this tendency (see Figure 5D). This may be a random result, as the group was small in number, but it may also be hypothesized that exposition to trauma divided the experimental group into two subgroups; individuals more susceptible to chronic trauma (less time in an Interaction Zone) and those more resilient (more time in an Interaction Zone). This requires further verification with a larger group, but if confirmed, that would imply that SIT shows individual variability in reactivity to stress induced by predator odor.

### Limitations

There are components of our research that should be expanded. As discussed earlier, a categorization is often applied in human studies of the subject, where individuals are characterized as stress-susceptible or resilient. We believe that our protocol could benefit if such a distinction was applied. A viable possibility may be the Avoiders/Non-Avoiders distinction proposed by Albrechet-Souza and Gilpin (2019) in their animal model of PTSD, or a hypothesized distinction delivered by SIT, as we discussed in paragraph above. While rats were in PST chambers, we did not videotape their activity. This is why we could not provide behavioral data from that time-period, that might have been interesting. Our conclusions regarding dopamine-related VTA and VS activity need further verification by molecular studies in animal models. Furthermore, there are interesting reports on striatal activity heavily influenced by increased inflammatory biomarkers, in the context of trauma (Mehta et al., 2020). We believe that our protocol could be of use in further exploration of these topics.

We believe further studies with our protocol should apply an additional group of rats exposed to non-predator odor. This could validate our approach with bobcat urine as a stressor, and deliver much needed comparative context. Changes in rodent behavior could be explored in exposure to different odors, for example alpha-pinene or green leaf odor that are known to have stress-alleviating effects (Akutsu et al., 2003). Studies that use different odors to examine behavioral and cognitive changes are sparse, thus we hypothesize our protocol could be of use to study this subject. We believe this comparative context would deliver interesting results in the wide issue of rodents behavioral and cognitive performance analysis.

## Conclusion

We present our protocol that may be useful in assessing cognitive abilities in rodents. Rats performed PST voluntarily, when exposed to chronic trauma induced by predator odor. Performance in PST was measured before and after trauma in the same group of rats. Subjects obtained better results in PST before exposure to predator odor. Overall, the experimental group scored lower in PST compared to not-traumatized control. After exposure to chronic trauma, rats were less socially oriented in SIT, compared to the results obtained before the trauma protocol. Moreover, traumatized rats presented a bimodal tendency in time spent in an Interaction Zone with unknown rat, but due to a small number of animals tested, this result needs further verification.

## Data Availability Statement

The original contributions presented in the study are included in the article/supplementary material, further inquiries can be directed to the corresponding author/s.

## Ethics Statement

The animal study was reviewed and approved by the Local Ethics Committee for Animal Experiments, Hirszfeld Institute of Immunology and Experimental Therapy, Polish Academy of Sciences, Wrocław, Poland.

## Author Contributions

TB designed the research, carried out laboratory experiments, and wrote the manuscript with input from all authors. PK designed and constructed Testing Chambers. JD performed mathematical calculations and analyzed obtained data. BS delivered theoretical framework. DF provided critical feedback and helped shape the research.

## Conflict of Interest

The authors declare that the research was conducted in the absence of any commercial or financial relationships that could be construed as a potential conflict of interest.

## Publisher’s Note

All claims expressed in this article are solely those of the authors and do not necessarily represent those of their affiliated organizations, or those of the publisher, the editors and the reviewers. Any product that may be evaluated in this article, or claim that may be made by its manufacturer, is not guaranteed or endorsed by the publisher.

## Footnotes

1
https://docs.scipy.org/doc/scipy/reference/stats.html
